# The Morphological Spectrum of Papillary Renal Cell Carcinoma and Prevalence of Provisional/Emerging Renal Tumor Entities with Papillary Growth

**DOI:** 10.3390/biomedicines9101418

**Published:** 2021-10-09

**Authors:** João Lobo, Riuko Ohashi, Birgit M. Helmchen, Niels J. Rupp, Jan H. Rüschoff, Holger Moch

**Affiliations:** 1 Department of Pathology, Portuguese Oncology Institute of Porto (IPOP), R. Dr. António Bernardino de Almeida, 4200-072 Porto, Portugal; jpedro.lobo@ipoporto.min-saude.pt; 2 Cancer Biology and Epigenetics Group, Research Center of IPO Porto (CI-IPOP)/RISE@CI-IPOP (Health Research Network), Portuguese Oncology Institute of Porto (IPO Porto)/Porto Comprehensive Cancer Center (Porto.CCC), R. Dr. António Bernardino de Almeida, 4200-072 Porto, Portugal; 3 Department of Pathology and Molecular Immunology, ICBAS—School of Medicine and Biomedical Sciences, University of Porto (ICBAS-UP), Rua Jorge Viterbo Ferreira 228, 4050-513 Porto, Portugal; 4 Histopathology Core Facility, Faculty of Medicine, Niigata University, 1-757 Asahimachi-Dori, Chuo-Ku, Niigata 951-8510, Japan; riuko@med.niigata-u.ac.jp; 5 Division of Molecular and Diagnostic Pathology, Graduate School of Medical and Dental Sciences, Niigata University, 1-757 Asahimachi-Dori, Chuo-Ku, Niigata 951-8510, Japan; 6 Department of Pathology and Molecular Pathology, University Hospital Zurich, Schmelzbergstrasse 12, 8091 Zurich, Switzerland; BirgitMaria.Helmchen@usz.ch (B.M.H.); niels.rupp@usz.ch (N.J.R.); JanHendrik.Rueschoff@usz.ch (J.H.R.); 7 Faculty of Medicine, University of Zurich, Rämistrasse 71, 8006 Zurich, Switzerland

**Keywords:** histopathology, biphasic squamoid alveolar RCC, biphasic hyalinizing psammomatous RCC, papillary renal neoplasm with reverse polarity, thyroid-like follicular RCC

## Abstract

Renal cell carcinoma (RCC) represents a heterogeneous disease, encompassing an increasing number of tumor subtypes. Post-2016, the World Health Organization (WHO) classification recognized that the spectrum of papillary renal cell carcinoma is evolving and has long surpassed the dichotomic simplistic “type 1 versus type 2” classification. The differential diagnosis of pRCC includes several new provisional/emerging entities with papillary growth. Type 2 tumors have been cleared out of several confounding entities, now regarded as independent tumors with specific clinical and molecular backgrounds. In this work we describe the prevalence and characteristics of emerging papillary tumor entities in two renal tumor cohorts (one consisting of consecutive papillary tumors from a single institute, the other consisting of consultation cases from several centers). After a review of 154 consecutive pRCC cases, 58% remained type 1 pRCC, and 34% type 2 pRCC. Papillary renal neoplasm with reversed polarity (1.3%), biphasic hyalinizing psammomatous RCC (1.3%), and biphasic squamoid/alveolar RCC (4.5%) were rare. Among 281 consultation cases, 121 (43%) tumors had a dominant papillary growth (most frequently MiT family translocation RCCs, mucinous tubular and spindle cell carcinoma and clear cell papillary RCC). Our data confirm that the spectrum of RCCs with papillary growth represents a major diagnostical challenge, frequently requiring a second expert opinion. Papillary renal neoplasm with reversed polarity, biphasic hyalinizing psammomatous RCC, and biphasic squamoid/alveolar RCC are rarely sent out for a second opinion, but correct classification and knowledge of these variants will improve our understanding of the clinical behavior of renal tumors with papillary growth.

## 1. Introduction

Kidney cancer represents the 14th most incident cancer worldwide, with 431,288 new cases diagnosed in 2020, corresponding to an age-standardized incidence rate of 4.6 per 100,000. It is the 15th deadliest malignancy, with 179,368 deaths documented and an age-standardized mortality rate of 1.8 per 100,000. The incidence is predicted to increase worldwide, and so is mortality, with 605,726 new cases and 285,906 deaths estimated in 2040 [1]. Median age at diagnosis is 64 years, and incidence is higher in males and in developed Western countries with high income, which is likely linked to lifestyle and exposure to risk factors, such as smoking, obesity, hypertension and chronic kidney disease [2].

Kidney cancer is a heterogeneous disease; it comprises several malignancies, the vast majority corresponding to renal cell carcinomas (RCCs), but also including sarcomas and other rarer entities [3]. Among RCCs, around 75% represent clear cell RCC (ccRCC). Most clinical advances, as well as the search for predictive/therapeutic biomarkers aiming at improving patients’ outcomes, have focused on ccRCC histology. Non-ccRCC patients show poorer responses when treated with targeted therapies conceived for ccRCC patients [4]. In the era of precision medicine, there is a need for histology-specific biomarkers and targeted therapies [5]. This endeavor is complicated by the well-known intra-tumor heterogeneity of RCCs as even within the same histological subtype, several morphological patterns and features can be present [6] and different molecular alterations can be found [7,8]. 

In recent years, our understanding of the RCC spectrum has improved greatly; few cancers have witnessed such an expansion in subtyping, with the emergence of several independent entities, either morphologically or molecularly defined [9,10,11,12]. This is illustrated by the evolving World Health Organization (WHO) classifications, with the last Edition of 2016 considering emerging/provisional entities (such as RCC with (angio)leiomyomatous stroma or *ALK* rearrangement-associated RCC), for which, in the meantime, additional convincing evidence has been gathered [13]. On the eve of releasing a new WHO classification, additional entities are to be introduced, further reducing the share of cancers placed into the category “RCC unclassified” (currently reported to represent 2–6% of epithelial renal tumors) [14,15].

Papillary RCC (pRCC) represents the second most common variant of RCC (10–20%). Delahunt and Eble proposed to distinguish papillary type 1 and type 2 RCC two decades ago [16]. The morphology of these variants has been described in the 2004 WHO classification and molecular differences were reported [17]. Importantly, it has been long recognized that mixed patterns are rather frequent in well-sampled pRCCs [18]. Furthermore, papillary features/areas may be seen in many other entities now considered outside of the pRCC spectrum [19]. In recent years, several studies have reported new renal tumor entities, as a result of a dedicated review of large case series and recognition of specific architectural or cytological patterns, supported by specific immunostainings and molecular studies. Of relevance are many of these so-called “emerging entities” which show papillary features or are actually more appropriately considered variants of pRCC, thus considerably shortening the “pure” pRCC spectrum. These include neoplasms such as papillary renal neoplasm with reversed polarity (PRNRP), biphasic hyalinizing psammomatous RCC (BHP RCC), biphasic squamoid/alveolar RCC (BSA RCC), or thyroid-like follicular RCC (TLF RCC) [9,11,12,20]. The prevalence of these recently described entities is hard to estimate, since few case series are yet reported; it is likely that our understanding of these tumors will expand in the near future. 

In this work we revisited two kidney tumor cohorts, describing the prevalence of emerging/provisional entities with a particular focus on the evolving morphological spectrum of pRCC. Specifically, we discuss recently acknowledged entities (and the emerging ones), and others where evidence is still building as to whether they should belong within the spectrum of pRCC. 

## 2. Materials and Methods

Two consecutive cohorts of nephrectomies/tumorectomies were retrieved from the Department of Pathology database at the University Hospital Zurich. The first consisted of in-house cases diagnosed as “pRCC”, signed out between 1993–present (cohort #1); the second corresponded to consultation cases on renal masses, originating from several institutes across Switzerland and Germany, signed off by HM (2017–present, cohort #2). Cohort #1 contained 154 pRCC cases, while cohort #2 included 281 consultation cases. 

Histological material was available for all cases and was reevaluated. One tumor block per cm tumor diameter was embedded for all tumors in cohort #1. At least one FFPE tumor tissue block was available for all cases of cohort #2. Additional immunostainings and fluorescent in situ hybridization (FISH) were ordered when appropriate (including immunohistochemistry for CK7, PAX8, AMACR, CD10, CAIX, CK20, SDHB, FH, GATA3, cyclinD1, Melan-A and TFE3, and FISH for TFE3 and TFEB). For this, several two micrometer-thick sections were ordered from formalin-fixed paraffin-embedded blocks. Immunohistochemistry was performed in automated platforms as routine diagnostic practice. All details about antibodies, vendors, clones, dilutions and platforms are displayed in Table 1. Appropriate positive and negative controls were embedded in each slide as tissue micro-arrays. Overall, results were considered “negative” if <5% of cells were stained, “positive, focal” if 5% to 25% of cells were stained, and “positive” if >25% of cells were stained. FH and SDHB were performed to rule out FH-deficient RCC and SDH-deficient RCC. TFE3 and Melan-A immunohistochemistry were performed when morphology was suggestive of MiT family translocation RCC and was followed by FISH for confirmation. GATA3 was performed to confirm a PRNRP diagnosis. CyclinD1 and CK20 were performed for aiding in the diagnosis of BSA RCC and eosinophilic solid and cystic RCC (ESC RCC).

FISH for *TFE3* and *TFEB* was performed with break-apart probes TFE3 bap Xp11.2 (Zytovision, Bremerhaven, Germany) and TFEB bap 6p21.1 (Empire Genomics, Williamsville, NY, USA), as routinely conducted for diagnostic purposes, following all steps indicated in the manufacturer’s protocol. Briefly, slides were pretreated, left to hybridize with the probe and submitted to posthybridization processing. Intelli buffer (Abbott, Des Plaines, IL, USA) was added to the protocol for reducing hybridization time. A total of 100 tumor cells were counted; the cutoff of positivity for TFE3 or TFEB translocation was >5% break apart signals. TFEB amplification was defined as >10 fusion signals.

Tumors were revisited, focusing on identification of recently described and emerging renal tumor entities.

## 3. Results

**Figure 1:**
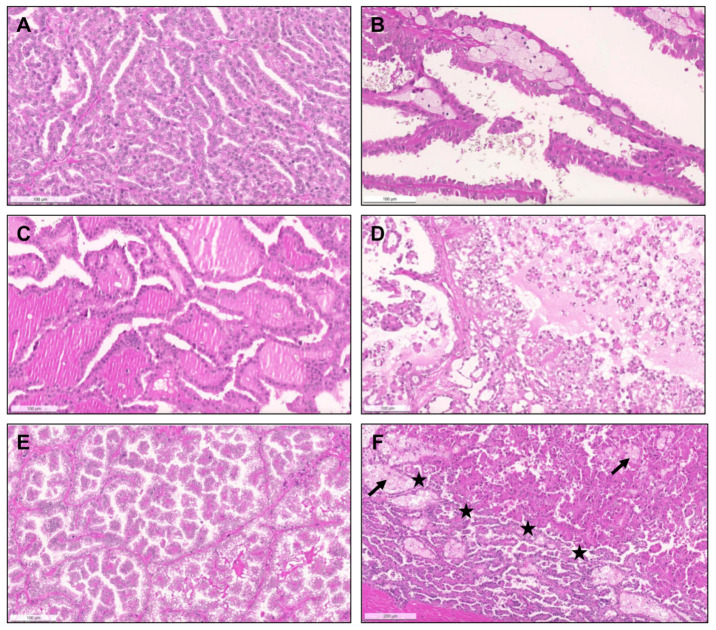
Heterogeneous histological patterns within pRCC. (**A**–**E**): (**A**) a case of a pRCC difficult to ascertain “a type”, due to the presence of multiple architectural patterns and cytological features within the same tumor, including packed elongated thin papillae filled by cells with small nuclei of lower WHO/ISUP grade; (**B**) elongated papillae lined by larger cells with eosinophilic cytoplasm, higher nuclei grade, pseudostratification of nuclei and hobnailing/apical snouts; (**C**) tubular/tubulocystic areas with small eosinophilic cells, filled with colloid-like material; (**D**–**E**) micropapillary images, with the presence of clear cells admixed with eosinophilic cells, dispersed within the stroma or within tubulopapillary structures; (**F**) a case of a pRCC showing an abrupt transition (stars) from a type 1 (classic) area, with thin papillae covered by small cells with pale cytoplasm and lower nuclear grade (bottom), to a type 2 area, with more dense papillae, covered by larger eosinophilic cells, with larger nuclei and nucleoli, and pseudostratification (top). Notice the typical xanthomatous macrophages common in pRCC (arrows).

**Figure 2:**
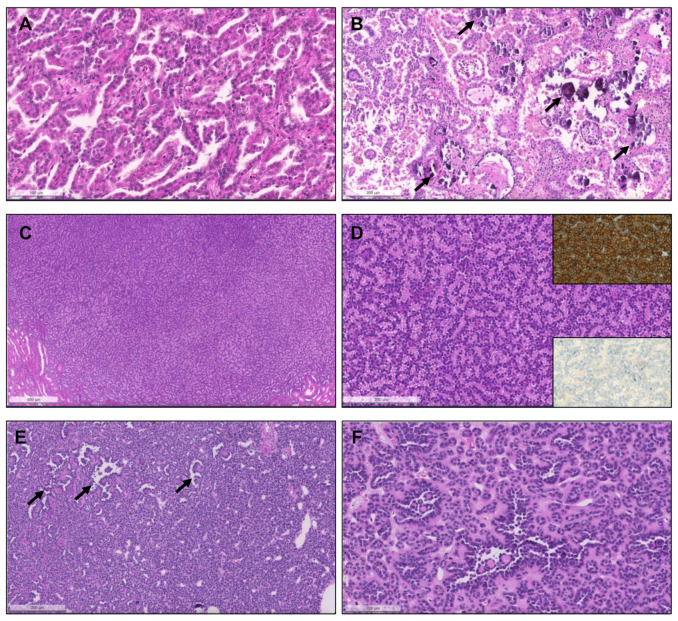
Patterns of pRCC type 1. (**A**) Classical features of pRCC type 1, with delicate papillae covered by cells with scant pale cytoplasm and nuclei arranged in a single layer. (**B**) Notice the frequent psammomatous calcifications (arrows) and macrophages filled with hemosiderin pigment; (**C**) Solid pattern of a pRCC in low power, mimicking metanephric adenoma. (**D**) At a higher power the papillary/tubulopapillary pattern is more evident, but is still challenging to distinguish from metanephric adenoma; (**E**,**F**) the correct diagnosis can be further confirmed with diffuse CK7 positivity (inset, upper right corner) and negativity for WT1 (inset, lower right corner). (**E**,**F**) Low and higher power aspects of a metanephric adenoma, showing a rather solid growth but with foci of papillary and tubular growth ((**E**,**F**), arrows) with psammomatous calcifications, raising concern for a pRCC. The tumor was, however, diffusely positive for WT1.

**Figure 3:**
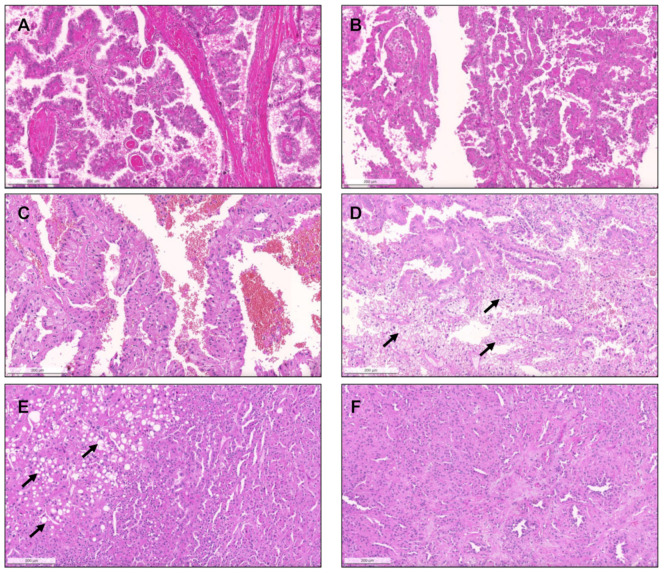
The spectrum of type 2 pRCC. Traditionally, these tumors are characterized by more complex papillae, covered by larger cells, with eosinophilic cytoplasm, usually of higher nuclear grade and with nuclear pseudostratification (**A**–**C**). Clear cells can be present ((**D**), arrows), as well as areas of vacuolation ((**E**), arrows). As for type 1 pRCC, a solid pattern can also occur, with packed papillae/tubules which are only discernible at high power magnification (**F**).

From 1993 until 2021 (cohort #1), a total of 154 pRCC diagnoses were consecutively made and histological material was revised, investigating the prevalence of specific new patterns/emerging entities recently described. Although classification of many tumors was difficult due to admixture of several architectural patterns and cytological features (Figure 1), most cases (57.8%) were regarded as type 1 pRCC (Figure 2), while 34.4% were regarded as type 2 pRCC (Figure 3).

**Figure 4:**
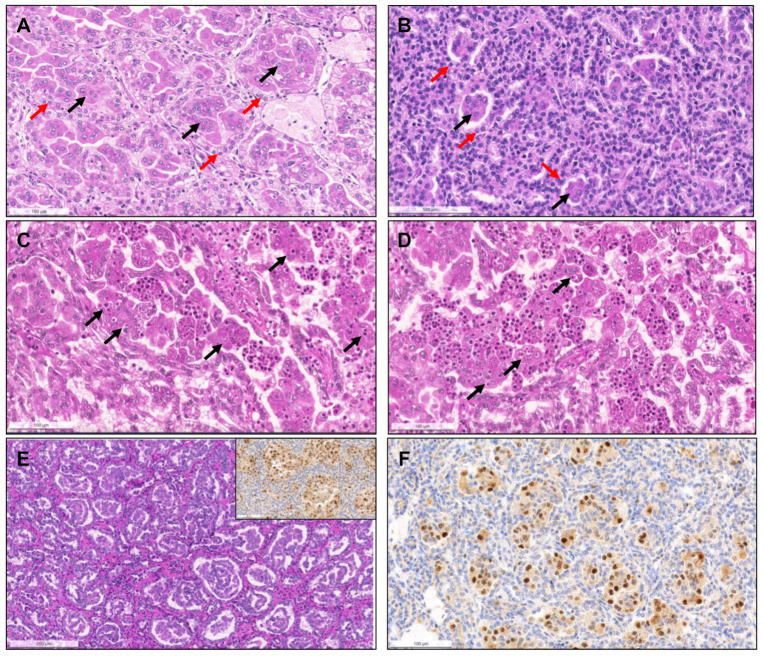
Biphasic squamoid/alveolar RCC pattern. Alveolar structures lined by smaller cells (red arrows) with scant cytoplasm and lower nuclear grade surround nests of larger cells (black arrows), with squamoid-like cytoplasmic features and higher nuclear grade, creating a biphasic and glomeruloid-like appearance, that was more prominent (**A**) or more discrete (**B**). Emperipolesis (engulfment of hematopoietic cells or parts of cells) were seen in most tumors with this pattern ((**C**,**D**), arrows). This pattern was also documented in a patient with a non-encapsulated tumor with less than 1.5 cm, meeting criteria for papillary adenoma (**E**). CyclinD1 immunoexpression was confirmed in all cases, restricted to the large cell population, highlighting them (inset in (**E**,**F**)).

**Figure 5:**
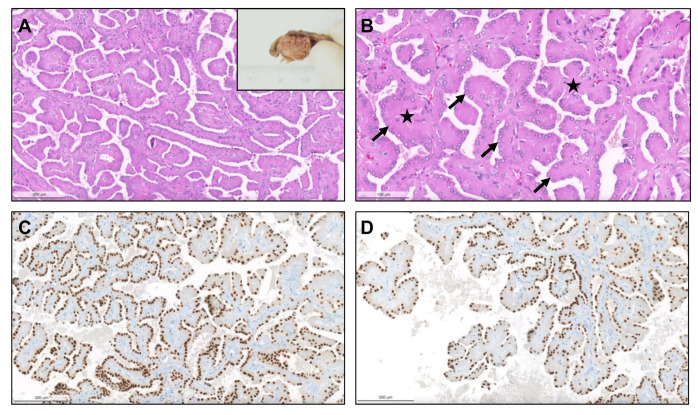
Papillary renal neoplasm with reversed polarity. The tumor was well demarcated, partly cystic and partly solid, yellowish and soft (inset). It was composed of small cells with oncocytic cytoplasm and small low-grade nuclei, displaced against the apical pole of the cells ((**A**,**B**), arrows). The papillary cores were hyalinized ((**B**), stars). The alignment of the nuclei “in a straight line” against the apical pole of the cells, lining the papillae contour, is further highlighted by GATA3, which is typically positive in these neoplasms (**C**,**D**).

**Figure 6:**
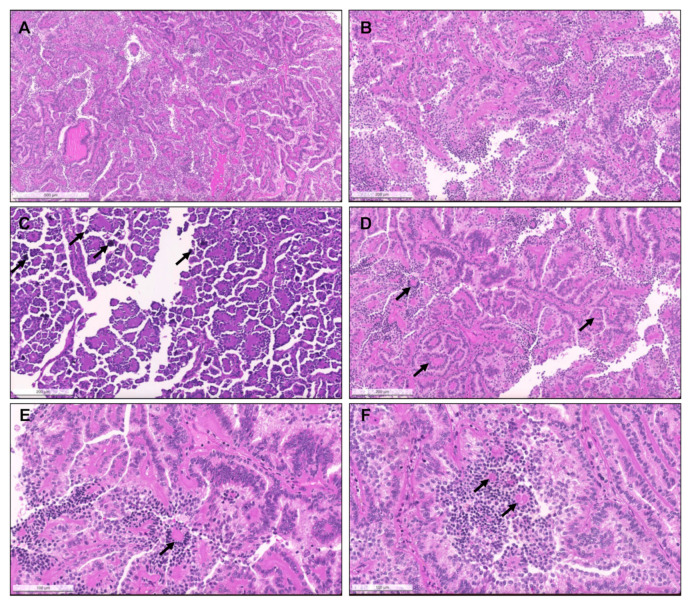
Biphasic hyalinizing psammomatous RCC. The biphasic nature of the tumor can already be seen at a low power (**A**). The tumor is composed of a population of small cells with small hyperchromatic nuclei, intermingled between and around a second population of larger cells. There is deposition of an eosinophilic basement membrane material (**B**). In some cases, the small cells were the predominant population, distributing around hyalinized papillae cores. Several small psammomatous calcifications were observed ((**C**), arrows). The larger cells cover the papillary fronds, and the smaller cells are tendentially distributed around basement membrane material, sometimes producing the aspect of pseudo-rosettes ((**D**–**F**), arrows).

Importantly, upon revision, seven tumors (4.5% of pRCCs) were identified that had a biphasic appearance, containing glomerular/alveolar structures lined by small cells with low nuclear grade, with squamoid-like larger cells in the center, with higher nuclear grade and specifically expressing nuclear cyclinD1; these were regarded as BSA RCC (Figure 4). Additionally, two (1.3% of pRCCs) small papillary tumors with oncocytic features and small low-grade nuclei aligned towards the apical pole of the cells were diagnosed as PRNRP, after confirmation of GATA3 nuclear immunoexpression (Figure 5). Although hyalinization and basement membrane material were focally found in some pRCCs, either in stroma or filling the papillary cores, only two cases (1.3% of pRCCs) showed a biphasic pattern with small cells disposed around basement membrane eosinophilic material and a second population of larger cells, as well as abundant small psammomatous calcifications, concordant with the diagnosis of BHP RCC (Figure 6). Only one tumor (0.7% of pRCCs) was found that histologically resembled thyroid parenchyma, despite being TTF1 and thyroglobulin negative, and was diagnosed as TLF RCC. No cases of Warthin-like pRCCs were documented.

A summary of the prevalence of papillary RCC in a single-institution cohort (cohort #1) is presented in Table 2.

**Figure 7:**
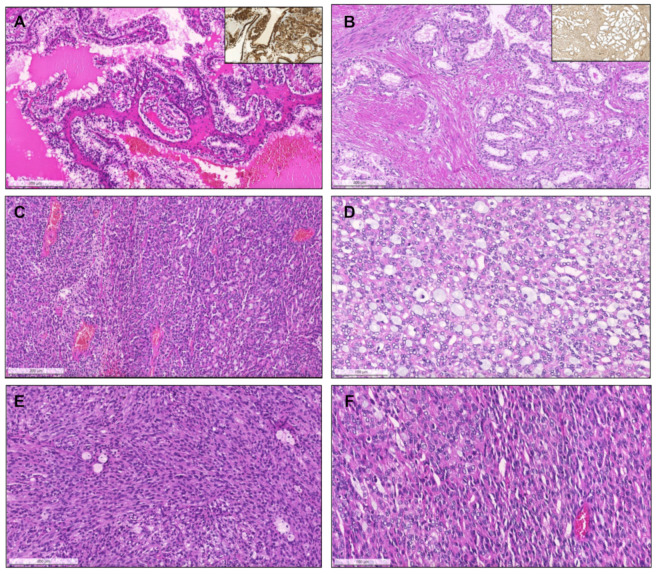
Clear cell papillary renal cell carcinoma, composed of papillae and tubulopapillary structures filled with clear cells, with small grade 1 nuclei, that show reversed polarity, distributed towards the apical pole of the cells. The tumors are diffusely positive for CK7 (inset, (**A**). Renal cell carcinoma with fibromyomatous stroma. Notice the remarkable smooth muscle fascicles surrounding and intersecting the tumor (positive for desmin, inset), which is composed mainly of groups of clear cells arranged in a tubule-papillary fashion (**B**). Mucinous tubular and spindle cell carcinoma. At low power (**C**), the transition between an area with compact tubular structures containing blue mucin (right) to an area with spindle/elongated cells is seen (left). In some cases, the tubular and mucinous features were remarkable, with the presence of mucin in the stroma and in the lumina of tubular structures (**D**), while in others the tumors were almost only composed of spindle cells, with elongated nuclei of low grade (**E**). Sometimes, the compact elongated tubules and stromal mucin are difficult to spot, only discernible at a higher power, resembling the solid pattern of pRCC (**F**).

**Figure 8:**
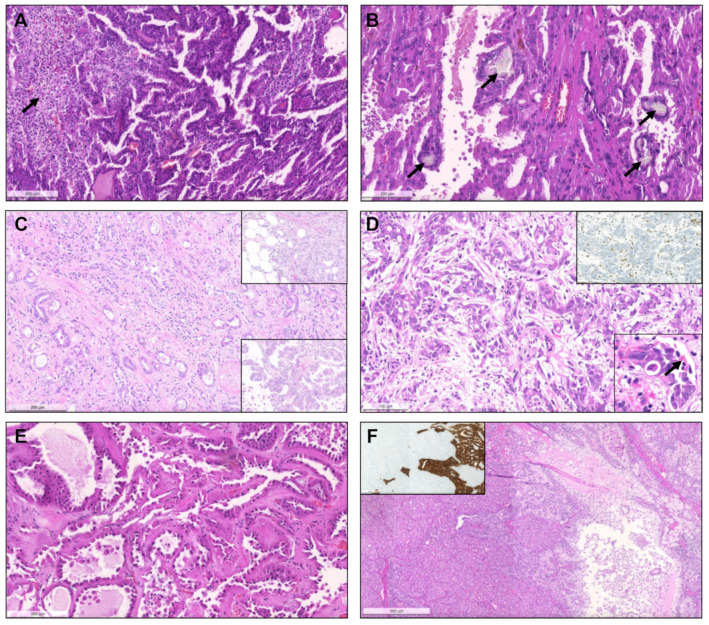
Acquired cystic disease-associated renal cell carcinoma, with prominent papillary fronds, intermingled with indistinct solid nodules of clear cells ((**A**), arrow) and with presence of the typical oxalate calcifications ((**B**), arrows). Collecting duct carcinoma, composed of highly infiltrative groups of cords and tubules disposed in a desmoplastic stroma (**C**). The tu-mor extensively infiltrated the hilar adipose tissue (inset, upper right corner). This case also showed papillary features focally (inset, lower right corner). SMARCB1 deficient medullary RCC, overlapping with collecting duct carcinoma (in-filtrative cords and tubules), with frequent angioinvasion, peritumoral neutrophils (**D**) and evidence of the characteristic sickled erythrocytes (inset, lower right corner, arrow). The tumor showed complete loss of INI1 immunoexpression (in-ternal positive control in adjacent lymphocytes and vessels). Tubulocystic renal cell carcinoma, being composed of tu-bulocystic structures filled by eosinophilic cells with prominent hobnailing and high grade nuclei, in a hypocellular fi-brotic stroma (**E**). A case of a collision tumor, with presence of a pRCC with classic morphology occurring in the middle of an oncocytoma (**F**). CK7 highlights the pRCC (inset).

**Figure 9:**
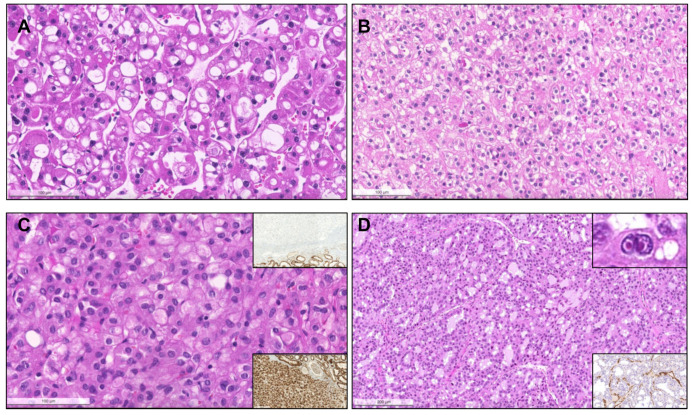
Eosinophilic vacuolated tumor of the kidney. The tumor is composed of cells arranged in small nests and cords, with eosinophilic cytoplasm and round nuclei with prominent nucleoli resembling oncocytoma, but the cytoplasm of tumor cells is remarkably vacuolated (small and large clear vacuoles) along the whole tumor (**A**). Succinate dehydrogenase deficient renal cell carcinoma. The tumor is classically composed of tubules and nests of mostly eosinophilic cells, with flocculent cytoplasm (**B**) and with vacuoles containing clear or slightly eosinophilic fluid, giving a bubbly appearance (**C**), but any morphology may be seen, including rare papillary features. The diagnosis is confirmed by the loss of expression of SDHB, with internal positive control in the adjacent renal tubules (inset, top right). Notice that SDHA expression is retained (inset, bottom right). Fumarate hydratase deficient renal cell carcinoma. The tumor showed a mixture of patterns, with solid, tubular, cystic and papillary areas (**D**). Several tumor cells presented the typical eosinophilic cytoplasm, round nuclei with prominent eosinophilic nucleoli surrounded by a clear halo (inset, top right), and showed the loss of cytoplasmic granular expression of fumarate hydratase in tumor cells (retained in infiltrating lymphocytes and in stromal vessels, inset, bottom right).

From 2017 until 2021, a total of 281 consults on renal masses were performed (cohort #2), and histological material was revised. Suspicion of a MiT translocation associated RCC in the presence of clear cells admixed with eosinophilic cells and papillary features, differential diagnosis of “pink tumors “and classification of predominantly papillary tumors with mixed or unusual patterns were the main reasons to send tumors for consultation. Out of 281 tumors in consultation, 121 had predominant papillary/tubulopapillary growth. The most frequent diagnosis rendered on consultation was ccRCC (58/281, 20.6%), followed by pRCC (56/281, 19.9%) and chromophobe RCC (chRCC, 48/281, 17.1%). In 17/56 pRCCs (30%) a distinction between type 1 versus 2 could not be made on the available material due to the mixture of several patterns and features of both types. Two cases with the presence of a biphasic pattern and containing a population of larger squamoid cells surrounded by smaller low-grade cells were compatible with BSA RCC. Additionally, two PRNRP were found. Important differential diagnoses of pRCC, such as clear cell papillary RCC (ccpRCC, *n* = 9), mucinous tubular and spindle cell carcinoma (MTSCC, *n* = 13), acquired cystic disease-associated RCC (ACD-associated RCC, *n* = 1), collecting duct carcinoma (*n* = 5), tubulocystic carcinoma (*n* = 1), SMARCB1 deficient medullary RCC (*n* = 1) as well as RCC with fumarate hydratase (FH) deficiency (*n* = 2) were found in this cohort (Figure 7, Figure 8 and Figure 9). Five collision tumors were diagnosed, three consisting of pRCC with oncocytoma, two consisting of ccRCC and pRCC.

Some solid renal tumors with eosinophilic cytoplasm can also show areas with papillary growth. Such tumor types include succinate dehydrogenase (SDH) deficient RCC, eosinophilic solid and cystic RCC (ESC RCC) and eosinophilic vacuolated tumor (EVT). Four cases of SDH deficient RCC were documented (Figure 9). Three eosinophilic tumors with solid and cystic areas were classified as ESC RCC and one fulfilled the criteria of EVT.

**Figure 10:**
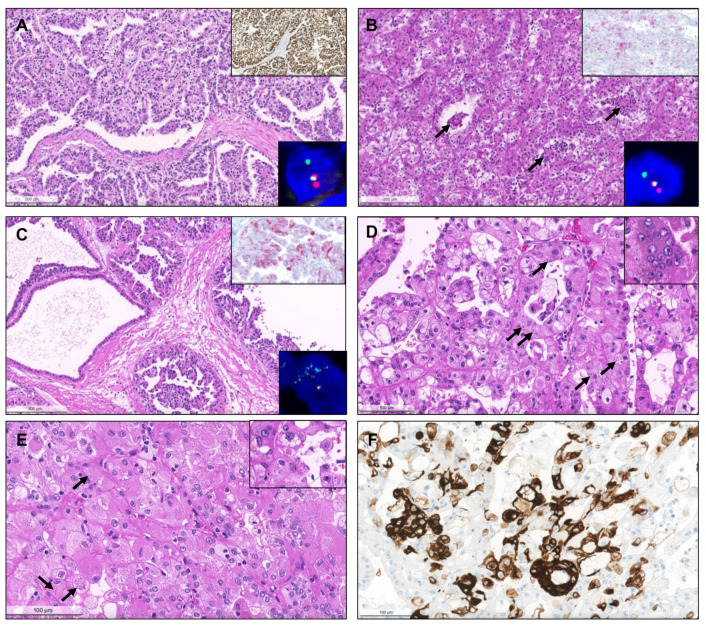
TFE3-translocated renal cell carcinoma. The tumor shows papillary architecture and clear cells (**A**) but can present with any morphology. Strong, diffuse positivity for TFE3 by immunohistochemistry strongly suggests the diagnosis (inset, right upper corner), which was confirmed by break-apart FISH (inset, right lower corner). TFEB-translocated renal cell carcinoma. Notice the admixture of clear cells and eosinophilic cells, also with the presence of a second population of smaller cells in clusters, focally surrounding or disposed within eosinophilic basement membrane material ((**B**), arrows). Positivity for Melan-A supports the diagnosis (inset, right upper corner), which was then confirmed by break-apart FISH (inset, right lower corner). TFEB-amplified renal cell carcinoma. The tumor showed a partly cystic, partly papillary architecture, with predominance of eosinophilic cells with prominent nucleoli (**C**). Melan-A was diffusely positive (inset, right upper corner) and the amplification was confirmed by FISH (inset, right lower corner). Eosinophilic solid and cystic renal cell carcinoma. Both tumors represented in (**D**) and (**E**) were solid and cystic, but also showed areas with papillary projections. The tumor cells were densely eosinophilic, with focal small clear vacuoles, and the typical basophilic cytoplasmic inclusions (stippling) were easily found at high power magnification ((**D**), arrows). There were also multinucleated eosinophilic cells (inset). Notice that many tumor cells are very large and “puffy”, with granular eosinophilic cytoplasm, and many nuclei are eccentric (contrarily to oncocytomas, where they are mostly centered). The nucleoli were prominent in some tumor cells, and both basophilic and slightly eosinophilic cytoplasmic granular inclusions (arrows) were seen (**E**, highlighted in the inset). The tumors showed strong multifocal positivity for CK20 (**F**).

Among MiT family translocation RCC, 11 were identified as TFE3 translocated RCC, 6 as TFEB translocated RCCs and one TFEB-amplified RCC. Presence of *TFEB* amplification was confirmed by FISH (Figure 10). All TFEB-altered RCCs expressed melanocytic markers.

A summary of the composition of the consultation cohort (cohort #2) is available in Table 3.

## 4. Discussion

### 4.1. Classic Papillary RCC

Post 2016 WHO classification, several provisional/emerging entities with papillary growth have been proposed. In our consecutive RCC cohort from a single institution, about 60% of pRCC fulfill the “classic” diagnostic criteria of type 1 pRCC. While several novel tumor entities with a specific clinical and molecular background have been removed from type 2 pRCC, about 35% of pRCC can still be assessed as type 2 pRCC. Provisional/emerging entities (papillary renal neoplasm with reversed polarity, biphasic hyalinizing psammomatous RCC, and biphasic squamoid/alveolar RCC overall only represent around 7% of all pRCC). In this study, we analyzed prevalence and differential diagnosis of renal tumor types with papillary growth.

For more than two decades, pathologists have attempted to morphologically discriminate among two types of pRCC: type 1 and type 2 tumors. The 2016 WHO classification acknowledged this morphological distinction, albeit hinting at emerging evidence that molecular data suggest that type 2 pRCC consists of at least 3 different categories [21,22]. Classification problems relate to reproducibility of reporting type 1 versus type 2 morphologies, the possible inclusion of specific new entities with a distinct clinical course in the type 2 pRCC family, and the frequent event of pRCC with mixed features. In our consultation cohort (cohort #2), 17% of pRCC could not unequivocally be classified as type 1 or 2, because they showed features of both. Such tumors have been previously designated as type 3 pRCC, but this has not been implemented in the WHO classification [18]. One study aiming at quantifying the proportion of type 2 morphology within these mixed pRCCs, found no prognostic effect of such type 2 extent [23]. Interestingly, we and others have seen pRCC in the context of “collision tumors” present in the same lesion together with other RCCs or even oncocytomas [24].

Type 1 pRCC is overall characterized by papillary and tubular structures, with evidence of delicate/thin fibrovascular cores, lined by small/medium-sized cells, usually cuboidal, arranged in a single layer. These features have increasingly been recognized as the “classical” form of pRCC. Other common findings include foamy histiocytes, psammomatous calcifications and pigmentation due to hemosiderin deposition. Predominant solid growth due to fusion of papillae is not uncommon. The papillary architecture may almost be completely obscured, hampering identification of the tumor as a pRCC. This pattern poses an important differential diagnosis with metanephric adenoma (and also epithelial-predominant nephroblastoma [25]), as well as with the (rare) well-differentiated primary neuroendocrine tumors of the kidney, all of which could be found in our cohort [26]. WT1 and negativity for neuroendocrine markers, together with CK7/AMACR positivity, assure that the correct diagnosis of pRCC is made. Metanephric adenoma and solid pRCC may be rather similar morphologically; histopathological clues include the presence of a thick fibrous pseudo-capsule in pRCC and an overall higher nuclear-to-cytoplasmic ratio in metanephric adenoma [27]. 

The differential diagnosis between pRCC and MTSCC was a frequent reason to seek a second opinion. Mucinous secretion has been described within pRCC [28]. This pattern is challenging, since mucin may trigger the diagnosis of MTSCC, characterized by tubular structures, spindle cells and mucin (rather “myxoid” in appearance), all in quite variable degrees. CD10 may be of help, as it is frequently positive in pRCC and usually negative/focal on MTSCC. Challenging cases benefit from molecular analyses, since MTSCC are cytogenetically distinct from classical pRCC, as they do not show gains of chromosome 7 and/or 17 commonly seen in pRCC, which is the main reason for considering MTSCC as an independent entity to date [29,30]. 

Nuclear grade (as defined by WHO/International Society of Urological Pathology—ISUP) and tumor stage are independent prognostic parameters in multivariable analyses of pRCC, [31]. pRCC classically spans an overall spectrum of low-grade to high-grade tumors. Although type 2 pRCC have been found in some studies to be associated with higher WHO/ISUP grade, higher stage at diagnosis and worse patient outcome, including poorer survival [32], other large studies showed that the prognostic value is lost in multivariable analyses [33]. A recent meta-analysis concluded that type 2 morphology did not translate into worse survival outcomes, contrarily to tumor stage, WHO/ISUP grade and other architectural patterns [34]. With the clear recognition of molecularly defined RCC with papillary growth (e.g., FH-deficient RCC), it can be hypothesized that “type 1” and “type 2” tumors may actually represent progression of “true papillary RCCs from lower to higher grade disease” rather than being different tumor types.

### 4.2. FH Deficient RCC and Tubulocystic RCC

In our consultation case series, the primary diagnosis of FH deficient RCC and tubulocystic RCC was mostly type 2 pRCC. The most classical features described for FH deficient RCC are the presence of round nuclei with prominent, eosinophilic viral inclusion-like nucleoli, surrounded by a clear halo. Usually, this entity has a papillary architecture, with cells showing abundant eosinophilic cytoplasm [35]. Importantly, pathologists should have a low threshold for ordering auxiliary FH (and S-(2succino)cysteine-2SC) immunohistochemistry [36]. The diagnosis of FH-deficient RCC should trigger genetic analysis, since most cases are seen as hereditary leiomyomatosis and RCC (HLRCC) syndrome-associated RCC [37]. Clinical investigation should include a search for cutaneous and uterine leiomyoma, especially those with atypical/bizarre cytological features [37]. Importantly, FH deficient RCC can also be seen in sporadic cases [38] and more frequent use of FH immunohistochemistry will help to identify more of these cases.

The main differential diagnoses of FH-deficient RCC includes tubulocystic RCC and collecting duct RCC. Tubulocystic RCC is considered if a tumor is exclusively composed of typical tubulocystic structures, with flat or hobnailed cells, abundant eosinophilic cytoplasm and high-grade nuclei, disposed in a hypocellular fibrotic stroma [39], and with the expression of FH by immunohistochemistry/no evidence of molecular FH alteration.

### 4.3. Collecting Duct Carcinoma and SMARCB1 Deficient Medullary RCC

The collecting duct carcinoma and SMARCB1 deficient medullary RCC were rare tumors in our consultation cohort. Collecting duct carcinoma may cause diagnostic difficulties with pRCC and FH-deficient RCCs [40], but the usual pattern of tubular structures, infiltrative growth with desmoplasia and localization of these tumors in the renal hilus usually creates more challenges in the differential to urothelial carcinoma of the renal pelvis [41]. Medullary RCC has been regarded as a variant of collecting duct carcinoma in the 2016 WHO classification. Of note, SMARCB1/INI1 inactivation has been recently identified as a molecular hallmark of most medullary RCC. Therefore, they should be classified as SMARCB1 deficient medullary RCC, a highly aggressive tumor in young patients with a sickle cell trait (with hypoxia of papillae caused by this condition possibly linked to tumorigenesis) [42]. It comprises several histological patterns, including tubular and papillary growth similar to collecting duct carcinoma [43]. 

### 4.4. Clear Cell Papillary RCC and Acquired Cystic Disease-Associated RCC

Clear cell papillary RCC (ccpRCC) and acquired cystic disease-associated RCC (ACD-associated RCC) were primarily described as specific tumors in end-stage renal disease [44]. In the following years, it was recognized that ccpRCC also occurs in the sporadic situation. These tumors, also described in literature as “clear cell tubulopapillary RCC” [45], represent the 4th most common subtype of RCC (after ccRCC, pRCC and chRCC) [46]. They are frequently cystic (possibly raising differential diagnosis with multilocular cystic RCC, since they can present with only small papillary foci emerging from cystic walls [47]) and display papillary and tubular (tubulopapillary) architecture lined by small cells of low nuclear grade and clear/pale cytoplasm, also showing reversed polarity like PRNRP. The typical immunoexpression of CK7 in a diffuse manner, and the cup-like staining for CAIX together with negativity for AMACR and CD10 clinch the diagnosis. The entity does not harbor *VHL* or 3p alterations [47]; given the indolent behavior of ccpRCC, the upcoming WHO classification will potentially rename the entity “clear cell papillary renal cell tumor”. Diagnosis should be reserved for those tumors fulfilling all criteria, especially in poorly sampled specimens [48]. 

ACD-associated RCC was extremely rare in our cohort. These tumors show a wide range of morphologies, and one should not forget that other RCC subtypes may also occur in end stage renal disease [49]. Tumors are frequently papillary, emerging within the cysts (likely the precursors of these cancers), and show evidence of oxalate calcifications, a rather characteristic feature. Papillary fronds also tend to alternate with foci of indistinct clear cell nodules [50].

### 4.5. Mixed Epithelial and Stromal Tumors

A significant number of mixed epithelial and stromal tumors (MEST) was sent out for consultation. MEST may also display papillary projections and features, especially when epithelial-predominant. Thorough sampling is sometimes necessary to identify the characteristic estrogen receptor-positive stroma that points to the right diagnosis, together with clinical history and predominance in perimenopausal women [51]. 

### 4.6. Provisional/Emerging Renal Tumor Entities with Papillary Growth

Upon revisiting our cohorts, we identified three eosinophilic solid and cystic (ESC) RCCs. The diagnosis was confirmed by CK20 expression. ESC RCC is characterized by solid sheets of eosinophilic cells mixed with macro- or microcystic areas. Tumor cells (both in solid areas and those lining the cyst walls) show a voluminous, “puffy” eosinophilic cytoplasm and prominent nucleoli, sometimes with eccentric nuclei or with multinucleation. A frequent finding is basophilic inclusions (stippling) within the cytoplasm (representing endoplasmic reticulum), and also eosinophilic cytoplasmic inclusions resembling leishmaniosis [52]. Focal vacuolation and admixture with clear cells, as well as papillary features, are also frequently observed. ESC RCC adds to the spectrum of renal neoplasms associated with alterations in TSC genes and mTOR pathway, which may have consequences for the selection of specific targeted treatments (such as mTOR inhibitors) [52,53]. mTOR pathway activation leads to upregulation of cathepsin K and subsequently to frequent expression of melanocytic markers [54]. 

Papillary renal neoplasm with reversed polarity (PRNRP) falls within the classical spectrum of pRCC, having been designated in literature as “oncocytic low-grade papillary RCC” or even “type 4 pRCC” [55,56,57,58,59]. Analysis of our cohorts revealed only four cases. These tumors are characterized by thinly branching papillae, filled by a single layer of small cells which are oncocytic and have small, low-grade nuclei. Strikingly, nuclei are mostly aligned and driven away from the basement membrane, towards the cell apex (reversed polarity). Tumors are usually small and low stage, and in one study features commonly seen in classical pRCC such as necrosis, psammoma bodies, mitotic figures and hemosiderin were absent in all tumors [58]. Dedicated investigations reported that these low-grade tumors are consistently positive for GATA3. Comparison with pRCC with more classical features led to the identification of recurrent *KRAS* mutations as a molecular hallmark of these tumors [59]. Correct classification is of relevance, since clinical course is favorable. 

Biphasic squamoid/alveolar RCC (BSA RCC) represented almost 5% of all pRCC in our single institutional cohort. Low power examination may show a rather solid tumor, but closer observation of this pattern will reveal its biphasic nature of two cell populations organized in a glomeruloid/organoid fashion: one of small, lower grade cells with scant cytoplasm lining alveolar structures, and the second composed of larger cells, of higher nuclear grade and with squamoid cytoplasmic features, present inside such alveolar structures [60]. One peculiar feature appreciated at high power magnification is the frequent presence of emperipolesis. However, this has (rarely) been documented in other renal tumors (such as ccRCC with multinucleated giant tumor cells [61]) and is hence not a defining feature. Interestingly, the large cells exclusively show nuclear staining for cyclinD1 (and additionally CD57) [62]), another rather distinctive feature. As with PRNRP, gains of chromosomes 7 and 17 were documented, suggesting that these tumors belong to the pRCC spectrum [63]. The high frequency of *MET* alterations [64] and frequent multifocality may have clinical implications. Upon revisiting our series of pRCC, the finding of larger squamoid cells, either in clusters or as individual cells, surrounded by a smaller cell population creating a glomeruloid-like pattern was noticed in 7/155 (4.5%) pRCCs, and was confirmed by exclusive nuclear cyclinD1 staining in these large cells.

Biphasic hyalinizing psammomatous RCC (BPH RCC) is another recently reported variant of pRCC [65]. As with BSA RCC, BPH RCC shows two cell populations, small and large, that are also arranged in a glomeruloid fashion (also frequently with solid areas). CK7 preferentially stains large cells, and EMA small cells. Distinctive features include condensation of the small cell population around dense basement membrane material (a pattern also frequently observed in the RCCs associated with TFEB translocation, which is also commonly biphasic in morphology), sometimes forming small pseudo-rosettes; sclerotic background, also with basement membrane material; and abundant psammoma bodies. More cases need to be investigated to understand the clinical implications of this pattern, if any. Somatic *NF2* mutations are the most likely driver mutations of this entity. Again, upon reviewing our series, we found hyalinization of papillary cores and inter-tumor stroma with basement membrane deposition in other tumors with type 1 pRCC features, but sometimes without clear evidence of the biphasic population being striking in only two cases. We believe that more definitive criteria for this pattern should be established to diagnose this tumor in the absence of *NF2* mutation analysis.

Another recently described unusual variant of pRCC is the highly inflamed *Warthin-like pRCC* [66], which we could not identify in our cohorts. In these tumors the papillae and stroma are filled with a dense lymphocytic infiltrate, and tumor cells are notoriously oncocytic, with prominent nucleoli, resembling in all aspects a Warthin tumor of the parotid gland. The oncocytoma-like cytoplasm and nucleoli lining the papillae in a single layer brings this entity close to the so-called “oncocytic pRCC”, for which consensus is lacking on exact histological criteria [67]. Oncocytic change can actually be frequently observed in most RCCs, not only pRCC [68]. 

### 4.7. Molecularly Defined RCC with Papillary Growth

MiT family translocation RCCs have a characteristic papillary growth of cells with clear cytoplasm and represented one of the most frequent tumor subtypes in the consultation cohort. Papillary architecture, in some cases extensive, and the presence of eosinophilic cells (especially when admixed with clear cells) should raise concern for MiT family translocated RCCs. This family includes both RCCs harboring translocations of Xp11 (*TFE3*) and also the less common t(6;11) (*TFEB*) [69]. Although TFE3 (and TFEB) immunohistochemistry may be practical screening techniques for diagnosing MiT translocated RCCs in daily routine (with only strong, diffuse positivity in tumor cells being interpreted as positive, since physiological low levels of TFE3 may also be encountered in tumors and adjacent stromal cells), the gold-standard approach remains documenting specific translocations with break-apart FISH, especially because it is less susceptible to fixation issues in paraffin-embedded samples [70]. Classically, TFE3-translocated RCCs show a mixture of papillary and nested growth, with clear cells and frequent psammomatous calcifications, but a long list of heterogeneous features and aspects have already been described [69]. TFE3 fusions were actually pinpointed in alveolar sarcomas of the soft parts, which share many morphological features with these renal tumors [71]. Staining for pan-cytokeratins, which are negative in most translocated RCCs, as opposed to the other RCC subtypes, should raise suspicion of a translocation-associated RCC. MiT family translocation RCCs are common in young patients. These tumors represent about 50% of pediatric RCCs, compared to 1–4% of adult RCCs [72]. There seems to be some genotype-phenotype association, since different partners of *TFE3* may have distinctive features and clinical meaning (for instance, cystic appearance when the partner is *MED15* [73], more aggressive behavior when the partner is *ASPSCR1* or *ASPL*, and less when in the presence of *PRCC* [74,75]). 

In our consultation cohort, we have seen TFEB translocation as well as one TFEB-amplified RCC. TFEB-translocated RCC is typically described as biphasic, with larger epithelioid cells accompanied by a second population of smaller cells, around basement membrane material, reminiscent of Call-Exner bodies in granulosa cell tumors [76]. They frequently express melanocytic markers (Melan-A and/or HMB45) and cathepsin K [77]. However, this also raises the differential of epithelioid PEComas, sharing the same immunoexpression pattern, except being PAX8 negative and CD68 positive [78]. Importantly, a subset of TFE3-translocated RCCs also show expression of melanocytic markers [79], also linked to the specific fusion partner, and can also have a rather biphasic pattern, further complicating the distinction [80]. Recently, TFEB-amplified tumors were described, which are usually aggressive and also show Melan-A expression [81]. Cathepsin K emerged as a very sensitive and specific marker of the MiT family of RCCs (since *TFE3* and *TFEB* are transcription factors of the same family of MITF contributing to cathepsin K activation) [82]. 

In our cohort, two RCC with fibromyomatous stroma (RCC FMS) were found. It is likely that these cases represent previously reported *TCEB1* (*ELOC*) mutated RCCs [83,84]. Diagnosis of this entity requires molecular analysis, because these tumors show a relatively broad morphological pattern [85]. Typical features are smooth muscle bundles transecting the tumor and dividing it into nodules of clear cells, usually with voluminous cytoplasm, but focal papillary features are also observed [86]. Investigation of more cases is needed to determine the clinical course of these tumors in comparison with ccRCC.

In our consecutive series, only one so-called *thyroid-like follicular RCC* (TLF RCC) was diagnosed. Recently, *EWSR1*-*PATZ1* fusions have been reported in TLF RCC [87]. These tumors remarkably resemble thyroid follicles, lined by small cuboidal cells and containing colloid material, and are still negative for TTF-1 and thyroglobulin (distinguishing them from metastatic thyroid carcinomas). Since classical pRCC may also show focal areas of follicles filled with inspissated colloid-like material, TLF RCC falls in the broader differential diagnosis of pRCC [88]. 

ALK-translocated RCC is the prototype of a molecularly-defined RCC, because this tumor may show many morphological aspects [89,90]. Mucinous depots should trigger ALK immunohistochemistry and/or FISH in a case of “unclassified RCC” with an unusual morphology. ALK inhibitors such as entrectinib may be potential targeted treatments in these tumors [91]. 

### 4.8. Solid Renal Tumors Showing Areas with Papillary Differentiation 

Papillary/tubulopapillary structures may be discernible in all renal tumor types, including ccRCC, chRCC and even oncocytoma [19,92,93]. Some oncocytic tumors are difficult to separate from papillary RCC, because oncocytic cytoplasmic changes can be seen in pRCC, translocation RCC and FH-deficient RCC [94]. SDH deficient RCCs infrequently pose problems in differential diagnosis with pRCC [95], but papillary and tubular growth patterns have been previously described [96].

In our cohort, we did find one case corresponding to the emerging category eosinophilic vacuolated tumor (EVT). The tumor fulfilled all diagnostic criteria [97], being well-demarcated but non-encapsulated, with normal renal tubules and vessels at the periphery, and composed of nests of cells with oncocytic cytoplasm, round nuclei with prominent nucleoli, and a remarkably vacuolated cytoplasm (smaller and larger vacuoles) throughout the whole tumor area. The tumor showed focal CK7 positivity in individual cells, and was CD117 positive, CD10 positive and vimentin negative. It has been discussed that the solid variant of pRCC should be considered as a differential diagnosis of EVT, especially in cases with oncocytic cytoplasm [98].

## 5. Conclusions

**Figure 11:**
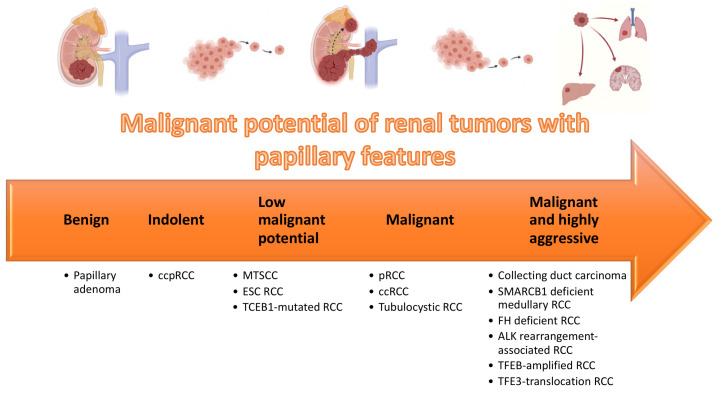
Organization of renal tumors with papillary features according to malignant potential.

RCC is a remarkably heterogeneous disease, with multiple subtypes. Recently acknowledged entities and patterns were reported, and their frequency is low. Centralized assessment of challenging renal tumors by dedicated uropathologists will contribute to improved knowledge of such entities. Integration of clinical, histological, molecular and topographical features, as well as background renal disease, is necessary for establishing the correct diagnosis, which may dictate patient prognosis, surveillance and guide further therapies. Renal tumors with papillary features (Table 4) represent a substantial proportion of cases sent for consultation. These include indolent tumors (e.g., ccpRCC), tumors with low malignant potential (e.g., MTSCC, ESC RCC) and highly aggressive tumors (e.g., collecting duct RCC and translocation RCC) (Figure 11). Novel targeted therapies will emerge that take advantage of the specificities of each tumor type and it seems insufficient to treat these tumors as non-clear cell RCC in clinical trials [99,100]. State of the art pathological evaluation, including recognition and description of clinically relevant features, is a fundamental cornerstone in the era of precision oncology. At the same time, as more entities are proposed, it is important that strict criteria are defined, allowing for investigation of pure cohorts of specific tumor entities.

## Figures and Tables

**Table 1:** Details about immunohistochemistry studies.

Antibody	Device	Dilution/Clone	Producer	Detection
AMACR	Ventana	1:30 [monoclonal] 13H4	Biologo	OptiView DAB
CAIX	BOND	1:3000 [polyclonal]	Abcam Limited	IHC Refine-30 min
CD10	Ventana	1:25 [monoclonal] 56C6	Novocastra Laboratories Ltd.	OptiView DAB
CK20	Ventana	Prediluted [monoclonal] SP33	Ventana-Roche	OptiView DAB
CK7	Ventana	1:100 [monoclonal] SP52	Abcam Limited	OptiView DAB
CyclinD1	BOND	1:40 [monoclonal] SP4	Labvision	IHC Refine-30 min
FH	BOND	1:1000 [monoclonal] J-13	Santa Cruz Biotechnology, Inc.	IHC Refine-30 min
GATA3	Ventana	1:200 [monoclonal] L50-823	Cell Marque Lifescreen Ltd.	OptiView DAB
Melan-A	Ventana	1:30 [monoclonal] A103	DAKO A/S	UView mono AP
PAX8	Ventana	1:100 [monoclonal] SP348	Abcam Limited	OptiView DAB
SDHB	BOND	1:200 [monoclonal] 21A11AE7	Abcam Limited	IHC Refine-30 min
TFE3	Ventana	Prediluted [monoclonal] MRQ-37	Cell Marque Lifescreen Ltd.	OptiView DAB

**Table 2:** Prevalence of papillary RCC in a consecutive single-institution cohort (cohort #1) after exclusion of 2016 WHO classification-recognized RCC types (e.g., translocation family RCC, ccpRCC, unclassified RCC, MTSC RCC, FH-deficient RCC and others).

Renal Tumor Subtype	*n* (%)
*pRCC*	
*type 1 (classic)*	89 (57.8)
*type 2*	53 (34.4)
*papillary renal neoplasm with reversed polarity*	2 (1.3)
*biphasic squamoid/alveolar*	7 (4.5)
*biphasic hyalinizing psammomatous*	2 (1.3)
*thyroid-like follicular*	1 (0.7)
*Warthin-like*	0
TOTAL	154 (100)

Abbreviations: pRCC—papillary renal cell carcinoma.

**Table 3:** Prevalence of renal tumor subtypes in a consultation cohort (cohort #2).

Diagnosis	N
ccRCC	58
chRCC	48
*of which*, *eosinophilic variant*	23
Oncocytoma	9
HOCT	2
EVT	1
SDH-deficient RCC	4
pRCC	56
*type 1 (classic)*	12
*type 2*	23
*mixed type 1/2*	17
*biphasic squamoid/alveolar*	2
*papillary renal neoplasm with reversed polarity*	2
ccpRCC	9
Acquired cystic disease-associated RCC	1
MTSCC	13
Multilocular cystic renal neoplasm of low malignant potential	2
Collecting duct carcinoma	5
SMARCB1 deficient medullary RCC	1
Tubulocystic RCC	1
FH-deficient RCC	2
ESC-RCC	3
MiT family translocation RCC	18
*of which*, *TFE3-translocated*	11
*of which*, *TFEB-translocated*	6
*of which*, *TFEB-amplified*	1
RCC with fibromyomatous stroma	2
MEST/cystic nephroma	6
Metanephric adenoma	1
Wilms’ tumor of the adult	1
Primary kidney NET, well differentiated	1
Collision tumor *	5
Angiomyolipoma	5
Angiosarcoma	1
Capillary hemangioma	1
Juxtaglomerular tumor	2
Liposarcoma	1
Synovial sarcoma	1
Epithelioid sarcoma	1
Myofibroblastic inflammatory tumor	1
Solitary fibrous tumor	1
Xanthogranulomatous pyelonephritis	1
IgG4 kidney disease	1
RCC, unclassified	16
TOTAL	281

Abbreviations: ccRCC—clear cell RCC; ccpRCC—clear cell papillary RCC; chRCC—chromophobe RCC; pRCC—papillary RCC; MEST—mixed epithelial and stromal tumor; MTSCC—mucinous tubular and spindle cell carcinoma; ESC RCC—eosinophilic solid and cystic RCC; HOCT—hybrid oncocytic-chromophobe tumor; EVT—eosinophilic vacuolated tumor; NET—neuroendocrine tumor; RCC—renal cell carcinoma; SDH—succinate dehydrogenase; FH—fumarate hydratase. * includes 3 pRCC with oncocytoma and 2 pRCC with ccRCC.

**Table 4:** Simplified overview of the organization of categories of renal cell tumors with papillary growth.

Architecturally/Cytologically Defined	Molecularly Defined	Anatomically Defined	With Associated Diseases
ccRCC	TFE3-translocated RCC	Collecting duct carcinoma	Acquired cystic disease-associated RCC
ccpRCC	TFEB-translocated RCC		
	TFEB-amplified RCC		
pRCC:	ALK rearrangement-associated RCC		
*Classic (type 1)*	SMARCB1-deficient medullary RCC		
*Solid*	TCEB1-mutated RCC		
*Warthin-like*			
*BSA RCC*			
BPH RCC *			
PRNRP *			
MTSCC			
ESC RCC			
Tubulocystic RCC			
TLF RCC *			

Abbreviations: BPH RCC—biphasic hyalinizing psammomatous RCC; BSA RCC—biphasic squamoid/alveolar RCC; ccRCC—clear cell RCC; ccpRCC—clear cell papillary RCC; ESC RCC—eosinophilic solid and cystic RCC; MTSCC—mucinous tubular and spindle cell carcinoma; pRCC—papillary RCC; PRNRP—papillary renal neoplasm with reversed polarity; RCC—renal cell carcinoma; TLF RCC—thyroid-like follicular RCC. * emerging renal tumors.

## Data Availability

All data is reported in the manuscript.

## Glossary

2SC	S-(2succino)cysteine
ACD-associated RCC	Acquired cystic disease-associated renal cell carcinoma
BHP RCC	Biphasic hyalinizing psammomatous renal cell carcinoma
BSA RCC	Biphasic squamoid/alveolar renal cell carcinoma
ccpRCC	Clear cell papillary RCC
ccRCC	Clear cell renal cell carcinoma
chRCC	Chromophobe renal cell carcinoma
ESC RCC	Eosinophilic solid and cystic renal cell carcinoma
EVT	Eosinophilic vacuolated tumor
FH	Fumarate hydratase
FISH	Fluorescence in situ hybridization
HLRCC	Hereditary leiomyomatosis and renal cell carcinoma
HOCT	Hybrid oncocytic-chromophobe tumor
ISUP	International Society of Urological Pathology
MEST	Mixed epithelial and stromal tumor
MTSCC	Mucinous tubular and spindle carcinoma
NET	Neuroendocrine tumor
pRCC	Papillary renal cell carcinoma
PRNRP	Papillary renal neoplasm with reversed polarity
RCC	Renal cell carcinoma
RCC FMS	Renal cell carcinoma with fibromyomatous stroma
SDH	Succinate dehydrogenase
TLF RCC	Thyroid-like follicular renal cell carcinoma
WHO	World Health Organization
